# Characterization of genotypes and antimicrobial resistance profiles of clinical isolates of *Shigella* from patients in the southern region of Iran

**DOI:** 10.1186/s40001-023-01570-0

**Published:** 2023-12-19

**Authors:** Saeed Shoja, Saba Ghasemi, Mahsa Dastranj, Jebreil Shamseddin, Nasim Ebrahimi, Hesam Alizade, Abbas Farahani

**Affiliations:** 1 Infectious and Tropical Diseases Research Center, Hormozgan Health Institute, Hormozgan University of Medical Sciences, Bandar Abbas, Iran; 2 Student Research Committee, Yasuj University of Medical Sciences, Yasuj, Iran; 3 Hepatitis and AIDS Department, Pasture Institute of Iran, Tehran, Iran; 4 Molecular and Medicine Research Center, Khomein University of Medical Sciences, Khomein, Iran; 5 Department of Medical Laboratory Sciences, Khomein University of Medical Sciences, Khomein, Iran

**Keywords:** Antibiotic resistance, *Shigella* species, Genetic diversity, ERIC-PCR

## Abstract

**Background:**

*Shigella* spp., which are facultative anaerobic bacilli within the *Enterobacteriaceae* family, present a significant public health burden due to their role as prominent contributors to diarrheal diseases worldwide. A molecular analysis can facilitate the identification and assessment of outbreaks involving this bacterium. So, we aimed to investigate the antibiotic susceptibility pattern and clonal relatedness of clinical *Shigella* spp. isolates obtained from patients with diarrhea in Hormozgan province, South of Iran.

**Methods:**

From 2019 to 2021, a cross-sectional investigation was conducted on 448 stool samples obtained from patients who were experiencing diarrhea, in the southern region of Iran. *Shigella* spp. isolates were identified based on biochemical and serological tests. All *Shigella* species were verified using species-specific polymerase chain reaction (PCR), followed by susceptibility testing to antimicrobial agents. Subsequently, genotyping of all *Shigella* species was conducted using ERIC-PCR.

**Results:**

Out of a total of 448 stool samples, the presence of *Shigella* was detected in 62 cases, accounting for a prevalence rate of 13.84%. Among the identified isolates, the majority were attributed to *S. flexneri*, representing 53.23% of the cases. This was followed by *S. sonnei* at 24.19% and *S. boydii* at 22.58%. Notably, no instances of *S. dysenteriae* were found. The highest prevalence of *Shigella* isolates was observed in infants and children under the age of five. A significant proportion of the identified isolates demonstrated resistance to various antibiotics. Specifically, high resistance rates were noted for ampicillin (90.78%), piperacillin–tazobactam (87.1%), cefixime (83.87%), trimethoprim–sulfamethoxazole (83.87%), cefotaxime (82.26%), and ceftriaxone (80.65%). In addition, a substantial number (87.1%) of the isolates exhibited a multidrug-resistant (MDR) phenotype. Using the ERIC-PCR method, a total of 11 clusters and 6 distinct single types were identified among all the *Shigella* isolates.

**Conclusion:**

A notable occurrence of antibiotic-resistant *Shigella* species has been noted, with multi-drug resistant (MDR) strains presenting an increasing challenge for treating shigellosis worldwide, and this includes Iran. Techniques such as ERIC-PCR are useful for assessing the genetic variation and connections between *Shigella* strains, which indirectly contributes to understanding antimicrobial resistance patterns. Further research is needed to explore the specific correlation between resistance genes and ERIC genotyping patterns in *Shigella* strains.

## Background

*Shigella* spp. the members of *Enterobacteriaceae* family are rod-shaped, non-spore-forming, non-motile, and facultative anaerobic Gram-negative bacterium that is capable of intracellular infection [1]. *Shigella* species are a major public health concern as they rank high among the causes of diarrhea across the globe. Ninety-nine percent of the roughly 167 million yearly instances of *Shigella* infection occur in developing nations [2]. Compared to other enteric pathogens such as *Salmonella* and *Vibrio* which typically require a dose of 10^5^–10^8^ organisms, *Shigellosis* poses a severe public health threat due to its infective dose being as low as 10 to 100 organisms [3]. There are four subgroups of *Shigella* spp., consisting of group A (*Shigella dysenteriae*), group B (*Shigella flexneri*), group C *(Shigella boydii*), and group D (*Shigella sonnei*), with each subgroup containing multiple serotypes [4].The use of antibiotics has resulted in benefits for patients infected with Shigella, including reduced duration and severity of diarrheal illness. Based on current guidelines from the World Health Organization (WHO) and a systematic review, the recommended treatment for Shigellosis is the administration of ciprofloxacin and azithromycin, two recommended oral antibiotics, for a duration of 5 days [5, 6]. Typically, shigellosis clears up without intervention or treatment, yet it can be dangerous for people with immunocompromised status those without adequate healthcare. *Shigella* can impact anyone, with children under five especially at risk because of inadequate hygiene practices, underdeveloped immune systems, and lack of prior exposure.

Antibiotics play a vital role in lowering diseases and death by treating bacterial infections, yet their improper and excessive use in managing diarrhea is a factor in the growing issue of antibiotic resistance. There is an escalating worry about the resistance of intestinal pathogens, including *Shigella *spp.*, Enteropathogenic E. coli* (EPEC), *Vibrio cholerae*, and *Salmonella *spp. [3, 7, 8].

A systematic review and meta-analysis was conducted in Iran between 2008 and 2021, revealing the prevalence rates of resistance to ciprofloxacin, azithromycin, and ceftriaxone among *Shigella* spp. as 3%, 30%, and 28%, respectively. Drug resistance in *Shigella* spp. can manifest through diverse mechanisms including active efflux pumps expelling drugs, reduced cellular permeability, increased production of drug-modifying or drug-inactivating enzymes, and target mutation leading to modification [3]. These strains commonly exhibit resistance to multiple antibiotics, leading in increasing of morbidity. Therefore, there is a strong need for the surveillance and control of these strains [3, 9]. Examining both the phenotypic and genotypic antimicrobial resistance profiles of *Shigella* is of utmost importance for accurately identifying appropriate antibiotics for the treatment of shigellosis, particularly given the ongoing alterations in resistance profiles [10]. Molecular typing techniques have been used with increasing frequency in studies of the epidemiology *Shigella* spp. and also for a better understanding of the evolutionary relationships among clones. Gaining knowledge about the prevalent strains linked to human infections and their origins within various environments holds significance for enhancing our comprehension of this pathogen's epidemiology and addressing related issues. Consequently, precise and swift epidemiological typing is imperative for tracking the evolution of these bacterium strains [10–12]. One of the suitable tools for genetic analysis and genetic relatedness evaluation among bacteria, especially the *Enterobacteriaceae* family, is the ERIC-PCR method. The objective of this study was to analyze the antibiotic susceptibility patterns and clonal relationships among clinical strains of *Shigella* spp. isolated from patients with diarrhea in the Hormozgan province, located in the southern region of Iran.

## Materials and methods

### Sample collection and processing

A cross-sectional study was conducted between March 2019 and November 2021 at Shahid Mohammadi, the principal teaching hospital associated with Hormozgan University of Medical Sciences in Hormozgan, Iran. The study focused on patients presenting with diarrhea who were referred to the hospital. Participants who satisfied the inclusion criteria, which consisted of a history of fever, cramps or abdominal pain, diarrhea, and vomiting, were included in the study. Individuals who had taken antibiotics within 48 h prior to sample collection were excluded. Stool samples were collected using sterile plastic containers and promptly transported to the hospital's microbiology laboratory.

The samples were subsequently directly inoculated onto MacConkey and xylose lysine deoxycholate (XLD) agar (Merck, Germany) using sterile disposable inoculation loops. They were subjected to aerobic incubation at 37 ºC for a duration of 24 h. *Shigella* spp. were confirmed through the utilization of conventional biochemical assays for identification. Colonies that exhibited characteristics suggestive of *Shigella* (including colorless and small pale colonies on MacConkey agar, and a red and green appearance on XLD and HE media, respectively) were subjected to inoculation into Sulfide Indole Motility Agar (SIM), Triple Sugar Iron Agar (TSI), and Lysine Decarboxylase Citrate Agar (LDC) (Merck, Germany), and then subjected to the urease production test [13]. Presumptive *Shigella* isolates were serologically grouped using commercial *Shigella* polyvalent antisera against the four *Shigella* species via slide agglutination tests (Baharafshan Institute of Research and Development, Iran). The positive control and reference bacterial strains used in this study were *S. sonnei* ATCC25931, *S. dysenteriae* ATCC13313, *S. flexneri* ATCC29903, and *S. boydii* ATCC8700.

### Molecular confirmation of *Shigella* spp. by species‑specific PCR

DNA extraction was performed utilizing the QIAamp DNA Mini Kit, in accordance with the guidelines provided by the manufacturer (Qiagen NV, Venlo, the Netherlands). DNA concentrations and quality were evaluated using the NanoDrop Spectrophotometer PROMO (Thermo Fisher Scientific, Waltham, MA, USA).

The identification of all *Shigella* species was achieved through DNA sequencing of putative integrase for *Shigella* genus, hypothetical protein for *S. boydii*, wbgZ for *S. sonnei*, rfpB for *S. dysenteriae*, and rfc for *S. flexneri *as previously described [14, 15].

The PCR reactions were prepared with a total reaction volume of 20 µL as follows: 0.5 µL of each 10 pM primer, 10 µL of 2 × Master Mix (SinaClon, Iran), 3 µL (50 ng) of extracted DNA, and sterile deionized water to reach a final volume of 20 µL.

Amplifications were performed using a thermocycler (C1000 Touch, Bio-Rad) following this protocol: initial denaturation at 95 °C for 5 min, followed by 35 cycles of denaturation at 95 °C for 60 s, annealing for 30 s (temperature dependent on primer nucleotide sequences in Table 1), and extension at 72 °C for 30 s, with a final extension at 72 °C for 5 min. Finally, 5 µL of the PCR products were run to a 1.5% agarose gel (Merck Co, Germany), then subjected to staining with SYBR Safe DNA gel stain (Invitrogen), and DNA bands were observed and documented utilizing a UV transilluminator device (Uvidoc, Gel documentation system, Cambridge, UK).

**Table 1:** The primers used in this investigation

Target gene (primers)	Forward sequence	Reverse sequence	Annealing temperature (^0^C)	Expected size (pb)	References
*Putative integrase*	TCGCATTTCTCTCCCCACCACG	CCGGATGTGTCTCGGGCAATC	63	159	[15]
*Hypothetical protein*	GAGCACGGAAACAGAGAGCGCC	GGTGCGTTCTTCCGGTGTTCTG	63	240	
*rfpB*	TCTCAATAATAGGGAACACAGC	CATAAATCACCAGCAAGGTT	59	211	[14]
*wbgZ*	TCTGAATATGCCCTCTACGCT	GACAGAGCCCGAAGAACCG	60	430	
*rfc*	TTTATGGCTTCTTTGTCGGC	CTGCGTGATCCGACCATG	60	537	
*ERIC*	ATGTAAGCTC CTGGGGATTCAC	AAGTAAGTGACTG GGGTGAGCG	55	–	[18]

### Antimicrobial susceptibility tests

The antimicrobial susceptibility of *Shigella* species was evaluated using the disk diffusion method (Kirby–Bauer test) on Mueller Hinton agar (Merck, Germany), in accordance with the recommendations provided by Clinical and Laboratory Standards Institute (CLSI 2022) guidelines [16]. Following antibiotic disks were used: amikacin (AN/30 μg), chloramphenicol (C/30 μg), trimethoprim–sulfamethoxazole (SXT/1.25/23.75 μg), nalidixic acid (NA/30 μg), norfloxacin (NOR/10 μg), ofloxacin (OFX/5 μg), ceftriaxone (CRO/30 μg), cefotaxime (CTX/30 μg), ceftazidime (CAZ/30 μg), ceftizoxime (CT/30 μg), cefixime (CFM/5 μg), azithromycin (AZM/15 μg), cefoxitin (FOX/30 μg), Amoxicillin (AMX/25), piperacillin–tazobactam (100/10 μg) (Mast Diagnostics, Merseyside, UK).

The phenotype(s) of *Shigella* isolates were classified as MDR (multidrug-resistant) when they displayed “non-susceptibility to at least one agent in three or more antimicrobial categories” [17].

### Molecular typing of *Shigella* spp. isolates

ERIC-PCR typing of the clinical isolates using two oligonucleotide primers was done as described previously [18]. PCR amplification was performed using an initial denaturation step at 95 °C for 10 min, followed by 4 cycles at 94 °C for 5 min, 5 min at 40 °C, and 5 min at 72 and then followed by 30 cycles of denaturation at 94 °C for 1 min, annealing at 55 °C for 1 min, extension at 72 °C for 2 min and the last extension at 72 °C for 10 min. Comparison of ERIC-PCR banding patterns was performed using GelJ software version 2.0 [19].

### Clustering analyses

The DNA patterns obtained were analyzed and compared following the methods described previously [20]. To identify ERIC-PCR polymorphism, the TIFF images of each gel photograph were inverted and normalized using a 100-bp DNA ladder as a size marker. Clustering analyses of the ERIC-PCR band patterns were conducted using GelJ software version 2.0, with the unweighted-pair group method (UPGMA) and average linkage Pearson coefficient at a 2% tolerance. Genotypes were clustered together if their similarity coefficient was equal to or exceeded 90%. *Escherichia coli* (ATCC 25922) was used as an outgroup for phylogenetic analysis.

## Result

### Description of sample population

In this cross-sectional descriptive study, out of the 448 individuals experiencing acute diarrhea, 62 (13.84%) received a shigellosis diagnosis by assessing clinical manifestations and laboratory results.

Among the 62 *Shigella* isolates, a total of 35 (56.5%) were detected in males, whereas 27 (43.5%) were identified in females. The percentages of clinical symptoms are shown in Table 2.

**Table 2:** The results of clinical symptoms among *Shigella* spp. in this study

Clinical characteristic	*Shigella* spp. (%)
Fever	93.54
Vomiting	82.25
Bloody diarrhea	56.45
Abdominal pain	80.64
Hospitalization	3.22

Furthermore, after amplifying the genes specific to each genus, it became apparent that the majority of isolates belonged to *S. flexneri* (53.2%), followed by *S. sonnei* (24.2%) and *S. boydii* (22.6%). Notably, no *S. dysenteriae* was detected. The season distribution of *Shigella* isolates is shown in Table 3.

**Table 3:** The season distribution of Shigella isolates

	Season				Total
	Spring	Summer	Autumn	Winter	
Species					
* S. flexneri*	0 (0)	0 (0)	16 (48.49)	17 (51.51)	33 (53.23)
* S. sonnei*	3 (20)	0 (0)	9 (60)	3 (20)	15 (24.19)
* S. boydii*	1 (7.14)	0 (0)	7 (50)	6 (42.86)	14 (22.58)
Total	4 (6.45)	0 (0)	32 (51.62)	26 (41.93)	62 (100)

The most prevalent of *Shigella* isolates was observed in infants and children under 5 years old (16.13 and 40.32, respectively) with *S. flexneri* (50% and 44%, respectively) and *S. sonnei* (30% and 36%, respectively) being the most frequent species (Table 4). Furthermore, *S. flexneri* exhibited the most occurrence among children aged 5 to 10 years.

**Table 4:** Incidence of *Shigella* spp. according to different age groups within the examined population

*Shigella* spp.	*S. flexneri*	*S. sonnei*	*S. boydii*	Total isolates (%)
Age group				
0–1	5	3	2	10 (16.13)
1–5	11	9	5	25 (40.32)
5–10	9	1	4	14 (22.58)
10–20	3	2	2	7 (11.29)
20–30	0	0	0	0
30–40	0	0	0	0
40–50	3	0	1	4 (6.45)
50–60	0	0	0	0
60–70	2	0	0	2 (3.23)
> 70	0	0	0	0
Total (%)	33 (53.23)	15 (24.19)	14 (22.58)	62 (100)

### Antimicrobial resistance profiles

The current investigation displays the sensitivity and resistance levels of the samples to antibiotics, presented in Table 5 through percentages and numerical counts. The antimicrobial susceptibility testing (AST) results demonstrated that ampicillin resistance was observed in 90.78% of the isolated *Shigella* spp., followed by piperacillin–tazobactam (87.1%), cefixime (83.87%), trimethoprim–sulfamethoxazole (83.87%), cefotaxime (82.26%), and ceftriaxone (80.65%). Furthermore, *Shigella* isolates exhibited sensitivity to norfloxacin, cefoxitin, ofloxacin, and chloramphenicol.

**Table 5:** The results of antimicrobial susceptibility profile of 62 *Shigella* isolates

Antibiotics	*S. flexneri* (n = 33)			*S. sonnei* (*n* = 15)			*S. boydii* (*n* = 14)			Total isolates (*n* = 62)		
	Sensitive (%)	Intermediate (%)	Resistant (%)	Sensitive (%)	Intermediate (%)	Resistant (%)	Sensitive (%)	Intermediate (%)	Resistant (%)	Sensitive (%)	Intermediate (%)	Resistant (%)
Amikacin (AN/30 μg)	5 (15.15)	18 (54.55)	10 (30.30)	6 (40)	7 (46.67)	2 (13.33)	8 (57.14)	2 (14.29)	4 (28.57)	19 (30.64)	27 (43.55)	16 (25.81)
Ampicillin (AMX/25 μg)	0 (0.0)	0 (0.0)	33 (100.0)	1 (6.67)	0 (0.0)	14 (93.33)	1 (1.14)	0 (0.0)	13 (92.86)	2 (3.22)	0 (0.0)	60 (90.78)
Azithromycin (AZM/15 μg)	22 (66.67)	2 (6.06)	9 (27.27)	8 (53.34)	2 (13.33)	5 (33.33)	6 (42.86)	4 (28.57)	4 (28.57)	36 (58.06)	8 (12.9)	18 (29.04)
Ceftriaxone (CRO/30 μg)	8 (24.24)	0 (0.0)	25 (75.76)	1 (6.67)	0 (0.0)	14 (93.33)	3 (21.43)	0 (0.0)	11 (78.57)	12 (19.35)	0 (0.0)	50 (80.65)
Cefotaxime (CTX/30 μg)	3 (9.09)	4 (12.12)	26 (78.79)	1 (6.67)	0 (0.0)	14 (93.33)	3 (21.43)	0 (0.0)	11 (78.57)	7 (11.29)	4 (6.45)	51 (82.26)
Ceftazidime (CAZ/30 μg)	14 (42.42)	5 (15.15)	14 (42.43)	5 (33.33)	8 (53.34)	2 (13.33)	4 (28.57)	4 (28.57)	6 (42.86)	23 (37.1)	17 (27.42)	22 (35.48)
Ceftizoxime (CT/30 μg)	6 (18.18)	5 (15.15)	22 (66.67)	4 (26.66)	4 (26.66)	7 (46.67)	4 (28.57)	1 (7.14)	9 (64.29)	14 (22.58)	10 (16.13)	38 (61.29)
Cefixime (CFM/5 μg)	8 (24.24)	0 (0.0)	25 (75.76)	1 (6.67)	0 (0.0)	14 (93.33)	1 (1.14)	0 (0.0)	13 (92.86)	10 (16.13)	0 (0.0)	52 (83.87)
Cefoxitin (FOX/30 μg)	33 (100)	0 (0.0)	0 (0.0)	14 (93.33)	0 (0.0)	1 (6.67)	8 (57.14)	1 (7.14)	5 (35.72)	55 (88.71)	1 (1.61)	6 (9.68)
Chloramphenicol (C/30 μg)	24 (72.73)	1 (3.03)	8 (24.24)	14 (93.33)	1 (6.67)	0 (0.0)	12 (85.72)	1 (7.14)	1 (7.14)	50 (80.64)	3 (4.84)	9 (14.52)
Ciprofloxacin (CP/5 μg)	4 (12.12)	12 (36.36)	17 (51.52)	3 (20.0)	5 (33.33)	7 (46.67)	2 (14.29)	1 (7.14)	11 (78.57)	9 (14.52)	18 (29.04)	35 (56.45)
Nalidixic acid (NA/30 μg)	14 (42.43)	13 (39.39)	6 (18.18)	6 (40)	1 (6.67)	8 (53.34)	2 (14.29)	4 (28.57)	8 (57.14)	22 (35.48)	18 (29.04)	22 (35.48)
Norfloxacin (NOR/10 μg)	33 (100)	0 (0.0)	0 (0.0)	14 (93.33)	0 (0.0)	1 (6.67)	11 (78.57)	0 (0.0)	3 (21.43)	58 (93.55)	0 (0.0)	4 (6.45)
Ofloxacin (OFX/5 μg)	30 (90.91)	3 (9.09)	0 (0.0)	11 (73.33)	3 (20.0)	1 (6.67)	11 (78.57)	1 (7.14)	2 (14.29)	52 (83.87)	7 (11.29)	3 (4.84)
Piperacillin–tazobactam (100/10 μg)	3 (9.09)	1 (3.03)	29 (87.88)	0 (0.0)	1 (6.67)	14 (93.33)	2 (14.29)	1 (7.14)	11 (78.57)	5 (8.06)	3 (4.84)	54 (87.1)
Trimethoprim–sulfamethoxazole (SXT/1.25/23.75 μg)	2 (6.06)	4 (12.12)	27 (81.82)	1 (6.67)	1 (6.67)	13 (86.66)	2 (14.29)	0 (0.0)	12 (85.71)	5 (8.06)	5 (8.06)	52 (83.87)

Among the strains of *S. sonnei*, a significant proportion of 93.33% exhibited resistance to ceftriaxone, cefotaxime, cefixime, and piperacillin–tazobactam. Notably, all strains belonging to *S. flexneri* demonstrated resistance to ampicillin. For further insight into the antibiotic resistance patterns within the three *Shigella* stereotypes, please refer to Table 5.

Out of the 62 isolates, 54 (87.1%) strains exhibited a multidrug-resistant (MDR) phenotype. Out of the 54 isolates exhibiting multidrug resistance (MDR), 30 were classified as *S. flexneri*, 13 as *S. boydii*, and 11 as *S. sonnei*.

### Molecular characterization using ERIC-PCR

**Fig. 1:**
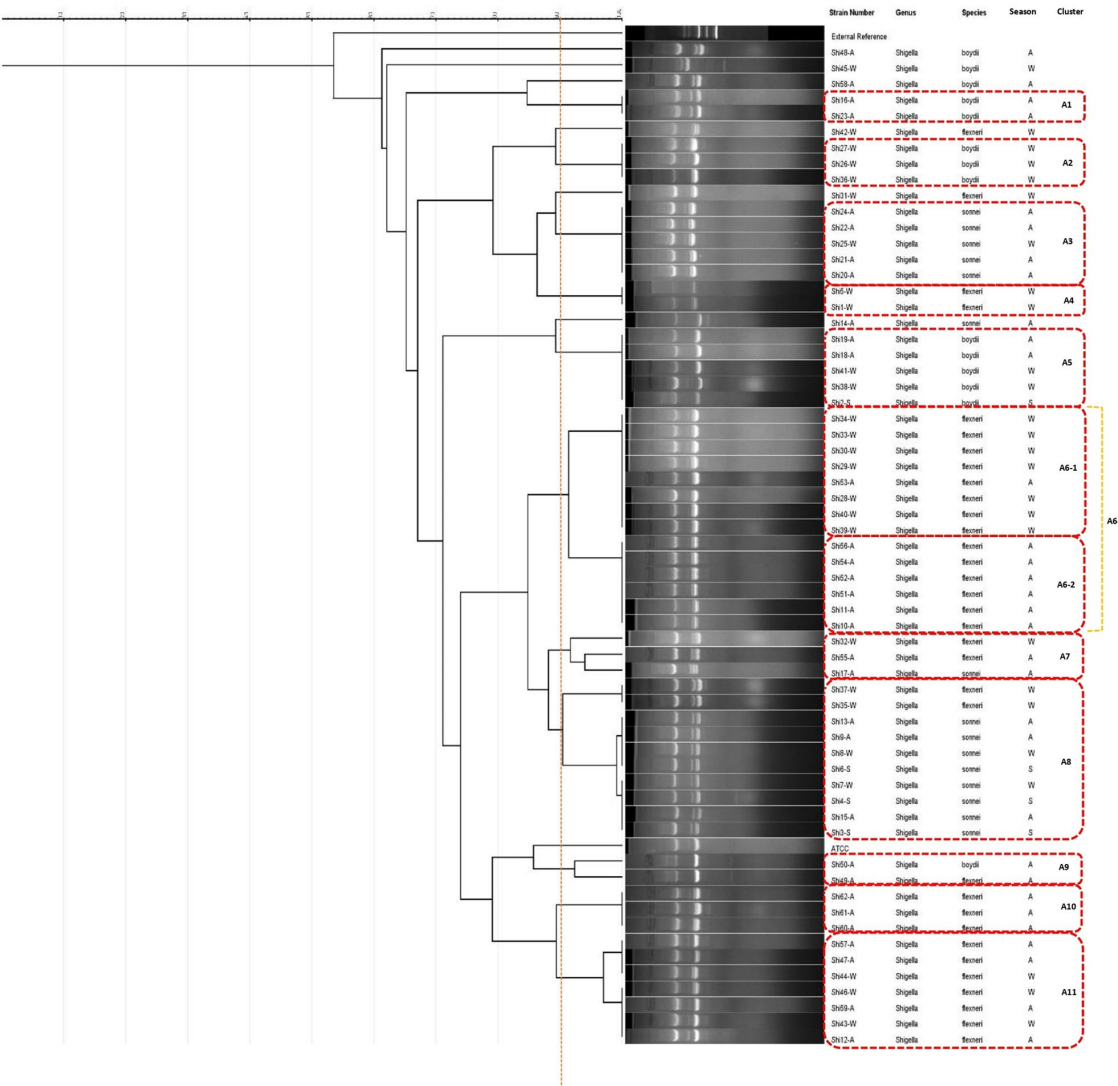
ERIC-PCR dendrogram of 62 *Shigella* spp. isolates in Hormozgan Province, Iran (Spring: S; Autumn: A; Winter: W)

The ERIC-PCR typing was performed to determine the genetic relatedness among *Shigella* isolates from stool samples in the South of Iran. All isolates were typed using the ERIC-PCR method. Among 62 *Shigella* isolates that were selected for molecular analysis, we identified 11 clusters, 2 sub clusters, and 6 distinct single types (Fig. 1).

Cluster A6 was found to be the predominant and pervasive cluster in our region, with a similarity rate of approximately 90–100%. Through the application of computer-assisted clustering, the strains demonstrated a substantial level of similarity, with similarity indices ranging from 85% for the most distinctive isolates to 100% for the indistinguishable ones. Interestingly, cluster A1, A2 and A5 contain only *S. boydii* isolates, whereas cluster A8 and A9 is relatively diverse. Clusters A4, A6, A10 and A11 only include strains *S. flexneri* and cluster A3 includes strains of *S. sonnei*.

The clusters A1, A3, A4, A6, A8, and A9 showed the highest prevalence of antibiotic-resistant isolates. For more information, please refer to Table 6.

**Table 6:** Frequency of antibiotic resistance profiles among different clusters

Clusters	A1	A2	A3	A4	A5	A6-1	A6-2	A8	A9	A10	A11
Antibiotics											
Ampicillin	2 (100)	3 (100)	5 (100)	2 (100)	4 (80)	8 (100)	6 (100)	9 (90)	2 (100)	3 (100)	7 (100)
Azithromycin	0 (0.0)	0 (0.0)	0 (0.0)	1 (50)	1 (20)	3 (37.5)	1 (16.7)	6 (60)	0 (0.0)	1 (33.3)	0 (0.0)
Ceftriaxone	2 (100)	2 (66.6)	5 (100)	2 (100)	3 (60)	8 (100)	5 (83.3)	9 (90)	2 (100)	1 (33.3)	3 (42.9)
Cefotaxime	2 (100)	2 (66.6)	5 (100)	2 (100)	3 (60)	8 (100)	5 (83.3)	9 (90)	2 (100)	2 (66.6)	3 (42.9)
Ceftazidime	1 (50)	2 (66.6)	1 (20)	1 (50)	2 (40)	6 (75)	3 (50)	0 (0.0)	2 (100)	0 (0.0)	2 (28.6)
Ceftizoxime	1 (50)	2 (66.6)	4 (80)	1 (50)	2 (40)	7 (87.5)	6 (100)	2 (20)	2 (100)	1 (33.3)	3 (42.9)
Cefixime	2 (100)	3 (100)	5 (100)	2 (100)	4 (80)	8 (100)	5 (83.3)	9 (90)	2 (100)	1 (33.3)	3 (42.9)
Cefoxitin	1 (50)	1 (33.3)	0 (0.0)	0 (0.0)	2 (40)	0 (0.0)	0 (0.0)	1 (10)	0 (0.0)	0 (0.0)	0 (0.0)
Chloramphenicol	0 (0.0)	0 (0.0)	0 (0.0)	1 (50)	0 (0.0)	3 (37.5)	2 (33.3)	0 (0.0)	0 (0.0)	1 (33.3)	1 (14.3)
Ciprofloxacin	2 (100)	3 (100)	4 (80)	0 (0.0)	4 (80)	3 (37.5)	2 (33.3)	3 (30)	1 (50)	1 (33.3)	6 (85.7)
Nalidixic acid	1 (50)	1 (33.3)	1 (20)	0 (0.0)	3 (60)	1 (12.5)	0 (0.0)	8 (80)	0 (0.0)	1 (33.3)	1 (14.3)
Norfloxacin	1 (50)	0 (0.0)	1 (20)	0 (0.0)	2 (40)	0 (0.0)	0 (0.0)	0 (0.0)	0 (0.0)	0 (0.0)	0 (0.0)
Ofloxacin	0 (0.0)	0 (0.0)	1 (20)	0 (0.0)	2 (40)	0 (0.0)	0 (0.0)	0 (0.0)	0 (0.0)	0 (0.0)	0 (0.0)
Piperacillin–tazobactam	2 (100)	3 (100)	5 (100)	2 (100)	4 (80)	8 (100)	5 (83.3)	9 (90)	1 (50)	3 (100)	5 (71.4)
Trimethoprim–sulfamethoxazole	2 (100)	3 (100)	3 (60)	2 (100)	3 (60)	7 (87.5)	3 (50)	10 (100)	2 (100)	2 (66.6)	7 (100)
Total	2 (100)	3 (100)	5 (100)	2 (100)	5 (100)	8 (100)	6 (100)	10 (100)	2 (100)	3 (100)	7 (100)

**Fig. 2:**
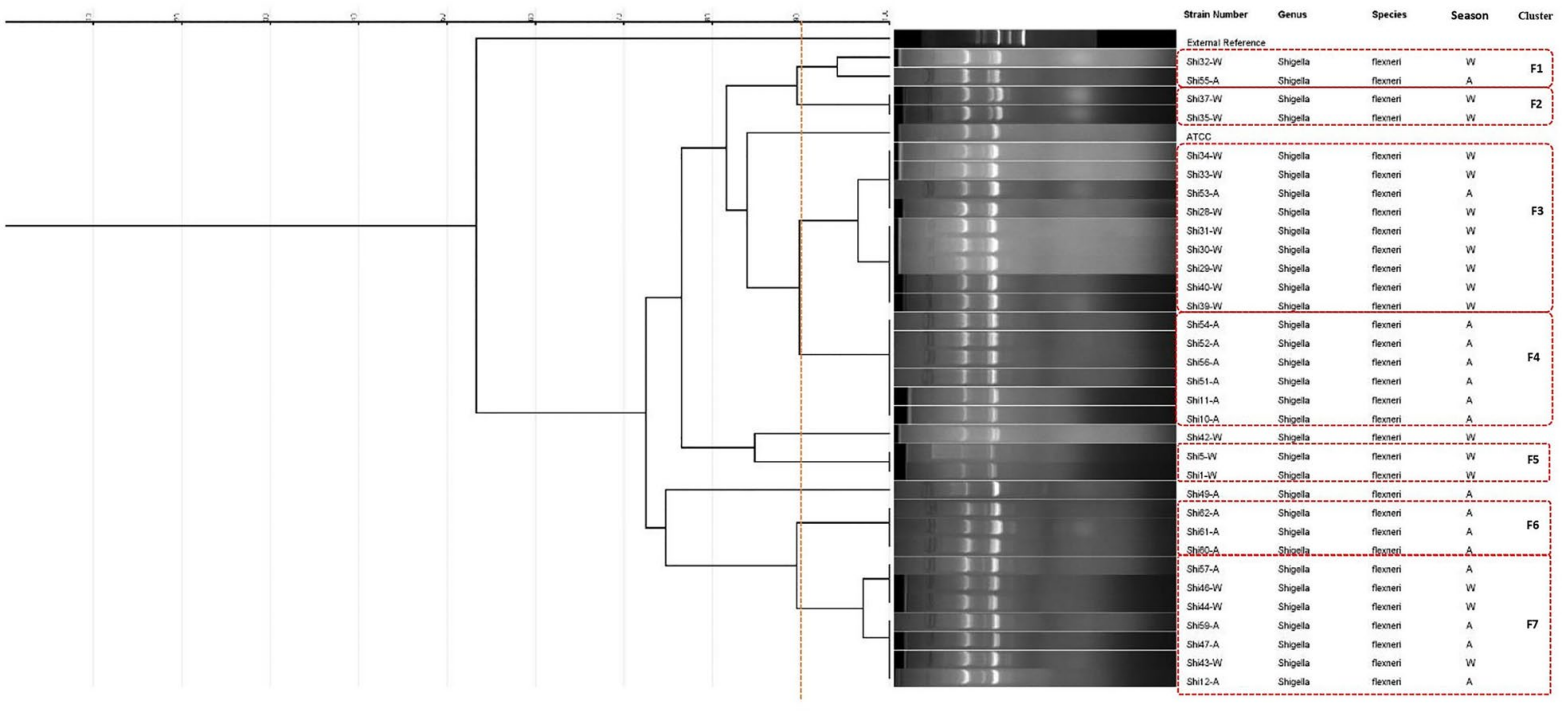
ERIC-PCR dendrogram of 33 *Shigella flexneri* isolates (Spring: S; Autumn: A; Winter: W)

**Fig. 3:**
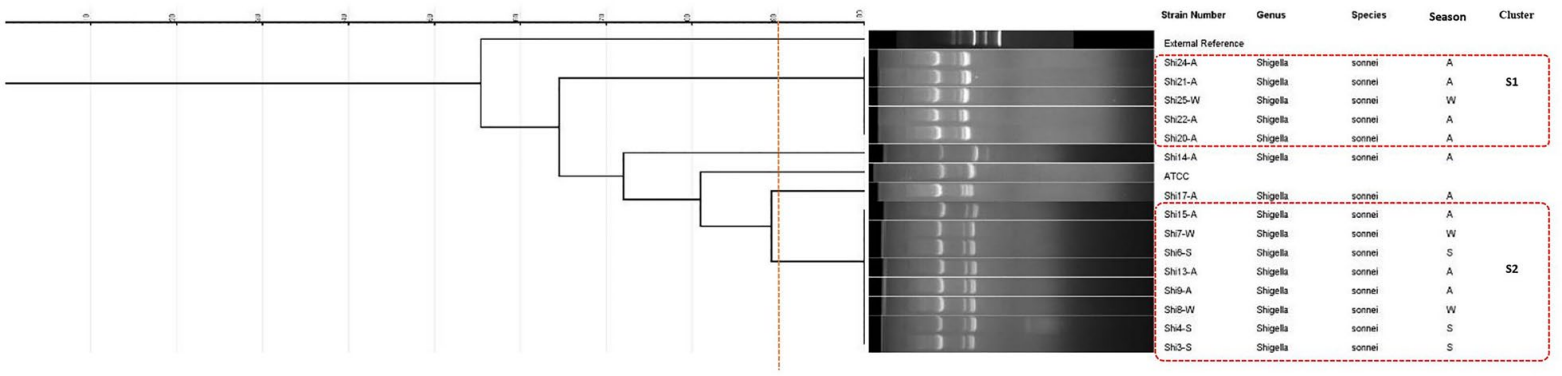
ERIC-PCR dendrogram of 15 *Shigella sonnei* isolates (Spring: S; Autumn: A; Winter: W)

**Fig. 4:**
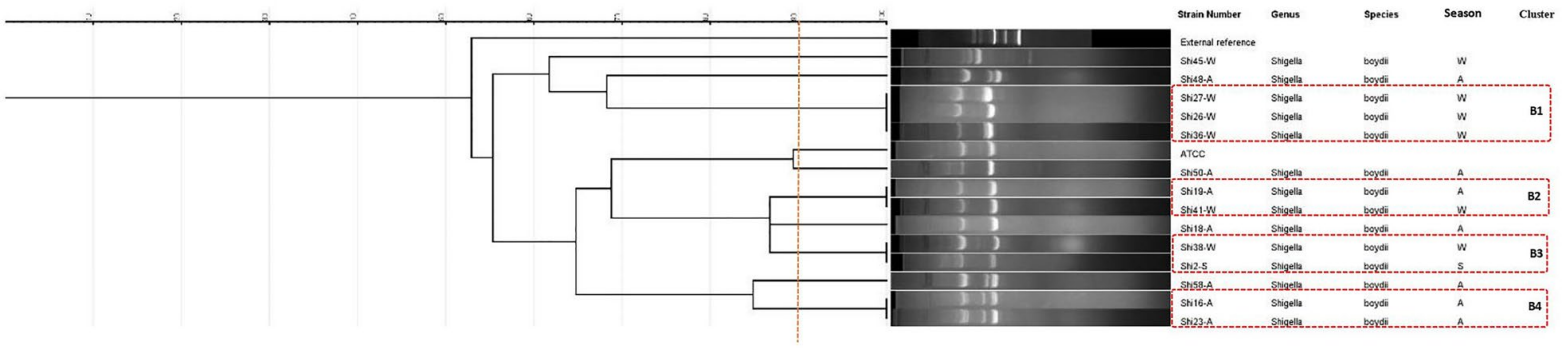
ERIC-PCR dendrogram of 14 *Shigella boydii* isolates (Spring: S; Autumn: A; Winter: W)

Individual analyses were conducted for each species, revealing that the *S. flexneri* isolates were categorized into seven clusters (F1 to F7) and two unique types (Fig. 2). The ERIC-PCR pattern analysis results for *Shigella sonnei* revealed the presence of two major clusters with 100% similarity among their isolates (Fig. 3). However, indicates that there is greater diversity among *S. boydii* isolates (Fig. 4).

**Fig. 5:**
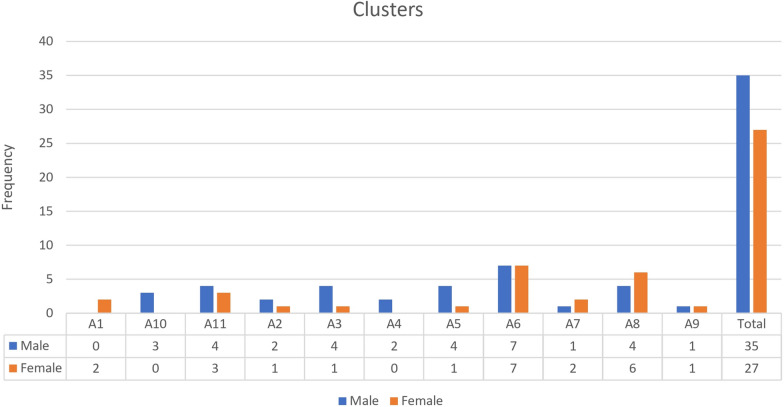
Distribution of different clusters of *Shigella* isolates among female and male patients

Clusters A4 and A10 are exclusively present in the male population, while cluster A1 is exclusive to the female population (Fig. 5).

## Discussion

Throughout the duration of our study, *S. flexneri* continued to maintain its dominance as the primary serogroup, consistent with previous records [3]. This research has confirmed the prevalence of *S. flexneri* in low- and middle-income countries (LMIC), while *S. sonnei* demonstrated a higher prevalence in high-income countries [21]. Therefore, the findings of this study validate the aforementioned assumption.

Over the past few decades in Iran, the predominant causative agent of shigellosis has been identified as *S. flexneri*. However, contemporary studies have revealed a notable surge in shigellosis cases associated with *S. sonnei* [22–24]. In this study, it was found that 40.3% of *S*. *flexneri* strains were isolated from children under 10 years of age, indicating a significant prevalence within this particular age group. The observed variations in the occurrence of *Shigella* spp. could potentially be attributed to factors such as age, economic development level, geographical location, climate, and numerous other environmental conditions [23].

*Shigella boydii* has been reported in Iran in less than 3% of the total shigellosis cases [9], whereas in our study, *S. boydii* accounted for 22.58%. In line with our study, research conducted in Iran in 2018 revealed that the occurrence rate of *S. boydii* among *Shigella* strains was 23.8% [25]. Similar to other studies in Iran, *S. dysenteriae* was not detected in stool samples [3, 23]. *Shigella dysenteriae* exhibits a higher frequency in outbreak scenarios linked to civil unrest and refugee crises [26].

In previous studies, it has been demonstrated that fever was the predominant presenting symptom [27, 28]. Our cases had a low rate of hospitalization and death was not observed. In contrast to previous research findings [29, 30], our study observed a high occurrence of bloody diarrhea (56.43%).

Various mechanisms contribute to the development of drug resistance in *Shigella* isolates. One example is the interference with DNA replication through the inhibition of DNA topoisomerase IV and gyrase by quinolone antibacterial agents, such as nalidixic acid, ofloxacin, and ciprofloxacin [3]. The emergence of antimicrobial resistance poses a critical challenge to public health. The results of antibiotic susceptibility testing revealed high resistance rates among *Shigella* isolates, particularly against ampicillin (90.78%), piperacillin–tazobactam (87.1%), cefixime (83.87%), trimethoprim–sulfamethoxazole(co-trimoxazole) (83.87%), cefotaxime (82.26%), and ceftriaxone (80.65%). These rates were in line with previous studies from Iran and various countries [3, 23, 31]. However, the resistance rates for different *Shigella* species exhibited the expected variations.

There are three subgroups of *Shigella spp.* isolates*: S. flexneri, S. sonnei,* and *S. boydii*. These subgroups have shown higher rates of resistance against ampicillin (64.9%) and co-trimoxazole. The data indicate a significant increase in resistance to various classes of antibiotics among the *Shigella* isolates. Based on our study findings, and considering that co-trimoxazole and ampicillin are commonly used as first-line treatments for *Shigella*-induced diarrhea, we discourage the use of these antibiotics for empirical therapy due to their high resistance rates [3]. In line with our findings, a significant resistance rate to different antibiotics had been previously reported in the southwestern and central regions of Iran [23, 32].

The resistance to ciprofloxacin has significantly risen (by 56.45%) when compared to previous studies [30, 33]. The occurrence of drug resistance in *Shigella* spp. is a significant risk, particularly in developing nations with health and nutritional challenges.

The findings of our study revealed a concerning trend with the high prevalence of MDR-*Shigella* isolates and their emergence within the respective hospitals. Similar to our results, a study conducted in Cambodia found that 91% of *Shigella* spp. isolates were multidrug-resistant [34].

In line with our study, a study conducted by Shahin et al. during 2015–2016 on 70 *Shigella* spp. isolates revealed that at least 50 percent of the *S. sonnei* strains were resistant to ceftazidime, cefotaxime, cefuroxime, ampicillin, tetracycline, nalidixic acid, and ciprofloxacin, according to the results of the antimicrobial [35].

A study conducted on *S. flexneri* and *S. sonnei* strains isolated from bacillary dysentery cases in Southeast Brazil found that 90% of them exhibited multiple drug resistance phenotypes [36].

Furthermore, across Iran, 11 studies identified a total of 667 clinical isolates as species producing both MDR and ESBLs [9]. There have been reports of the emergence of extensively drug-resistant (XDR) and multidrug-resistant (MDR) *S. flexneri* serotypes in England [37].

Similar to the isolates isolated from human sources, unfortunately, the isolates isolated from water sources have also shown high antibiotic resistance. For example, Shahin et al. from Iran showed that all *Shigella* isolates collected from water samples were MDR [38].

In a study with a wide range of food samples demonstrated that the incidence of *Shigella* spp. is more frequent in raw vegetables and also multidrug resistance phenotypes were noticeably frequent and observed in 17 isolates (89.5%) out of 19 isolates.

A study conducted on various food samples reveals a high prevalence of *Shigella* spp. in raw vegetables, with a remarkable prevalence of multidrug resistance phenotypes detected in 17 out of 19 isolates (89.5%). The spread of *Shigella* is notably affected by suboptimal health standards, limited personal hygiene awareness, and water quality of lower quality. Although most cases of Shigella-induced foodborne illnesses are typically mild and resolve independently, the emergence of severe cases raises significant concerns, particularly in high-risk patient populations [39].

In this current investigation, ERIC-PCR was utilized for the molecular characterization of *Shigella* spp. isolates, offering a swift and cost-effective approach that has demonstrated its worth as a valuable genotyping method for *Enterobacteriaceae* [40]. It is a powerful method for molecular typing of *Shigella* strains and has been shown to be a plausible alternative strategy to PFGE [41]. This study aimed to understand the molecular epidemiology of *Shigella spp*. in hospitals. The objective was to identify the main clonal lineages that are currently circulating in this region.

Isolates in this study with ERIC profiles A3, A5, A6, A8 and A11 were believed to be endemic in our study region. All isolates belonging to the same genotype exhibited consistent resistance patterns. They demonstrated co-resistance to antimicrobial agents, including ampicillin, piperacillin–tazobactam, trimethoprim–sulfamethoxazole, cefixime, and ceftriaxone (Table 6). This remarkably similar pattern suggests that the same species might be circulating within the communities. Also, based on the ERIC profile results of all isolates presented in Fig. 1, clusters A2, A4, and sub cluster A6-1 (87.5%) were prevalent in the winter season, while clusters A1, A3, A9, A10 and sub cluster A6-2 were prevalent in the autumn season. Maybe this reason is one of the factors contributing to the high similarity of these isolates.

Wei et al. documented a strong correlation between antimicrobial resistance patterns and PFGE genotypes in clinically derived *Shigella* isolates [42]. *Shigella* isolates with the same antimicrobial resistance patterns, as well as similar PFGE genotypes, have contributed to the shigellosis that has been circulating in an area for a longer period.

In another study conducted by Zhu et al., a notable correlation was observed between the antimicrobial resistance phenotypes and genotyping of *Shigella* spp. isolates obtained from clinical samples [43].

In our research, groups A1 and A9 showed complete resistance to seven different antibiotics, as detailed in Table 6. Corresponding with our results, another investigation used RAPD-PCR to group *Shigella* strains into five main clusters and found that those in cluster R4 exhibited the most antibiotic resistance. This study also suggested a connection between the clusters and their resistance to antimicrobials [44]. Therefore, molecular genotyping patterns can be used to predict antimicrobial resistance in *Shigella* strains.

In the last 20 years, numerous studies have applied the ERIC-PCR assay as a quick genetic typing technique to evaluate genetic connections between bacterial species from various origins such as the environment, food products, and humans [20, 45, 46]. In this study, ERIC-PCR showed remarkable differentiation among unrelated *Shigella* spp., making it a valuable DNA fingerprinting approach for discerning *Shigella* species isolated from diverse samples. It can provide genotyping profiles and contribute to understanding the genetic diversity and relatedness of *Shigella* strains.

## Conclusion

There has been a notable increase in *Shigella* strains resistant to antibiotics. The rise of multi-drug resistant *Shigella* is alarming for treating shigellosis worldwide, Iran included. This complicates the management of *Shigella* infections and underscores the urgency for measures to tackle antibiotic resistance. On the other hand, while there is limited specific information on the correlation between antimicrobial resistance and ERIC genotyping patterns in clinically derived *Shigella* strains, genotyping methods like ERIC-PCR can provide valuable information about the genetic diversity and relatedness of *Shigella* strains, which indirectly contributes to understanding antimicrobial resistance patterns. Further research is needed to explore the specific correlation between resistance genes and ERIC genotyping patterns in *Shigella* strains.

## Limitation

Our study possesses certain limitations. Firstly, we have not examined the resistance genes among isolates that are resistant to multiple drugs. Furthermore, information regarding the severity of the disease is lacking. Consequently, the association between disease severity and antibiotic resistance remains unexplored. Moreover, it is important to note that our study is restricted to a single-center design.

## Data Availability

All data generated and analyzed during the current study are available from the corresponding author on reasonable request.

## Abbreviations

MDR: Multidrug-resistant
XDR: Extensively drug-resistant
PCR: Polymerase chain reaction
ERIC-PCR: Enterobacterial Repetitive Intergenic Consensus Polymerase Chain Reaction
EPEC: Enteropathogenic *E. coli*
UPGMA: Unweighted-pair group method
LMIC: Low- and middle-income countries
